# Correction: Infectious polymorphic toxins delivered by outer membrane exchange discriminate kin in myxobacteria

**DOI:** 10.7554/eLife.37049

**Published:** 2018-04-05

**Authors:** Christopher Vassallo, Pengbo Cao, Austin Conklin, Hayley Finkelstein, Christopher Hayes, Daniel Wall

Vassallo CN, Cao P, Conklin A, Finkelstein H, Hayes CS, Wall D. 2017. Infectious polymorphic toxins delivered by outer membrane exchange discriminate kin in myxobacteria. *eLife* **6**:e29397. doi: 10.7554/eLife.29397.

Published 18, August 2017

During reorganizing Figure 1B for resubmission a duplicate image mistakenly replaced another image that was very similar. Specifically, under Colony phenotype the image in row 5 was inadvertently duplicated in the row below. We have now replaced the duplicated image with the originally intended image. This correction does not affect any result described by the figure.

The Corrected Figure 1B is shown here:

**Figure 1:**
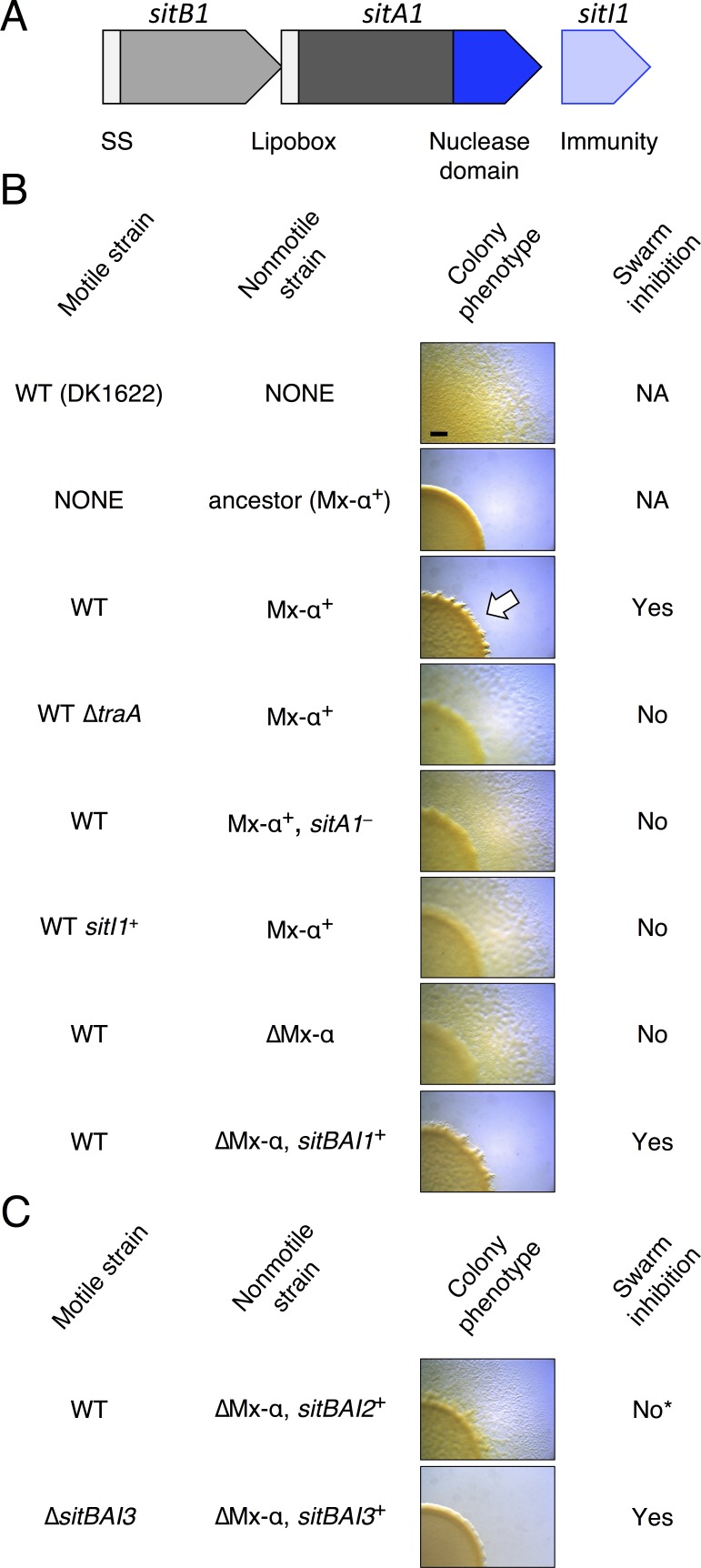


The originally published Figure 1B is also shown for reference:

**Figure 2:**
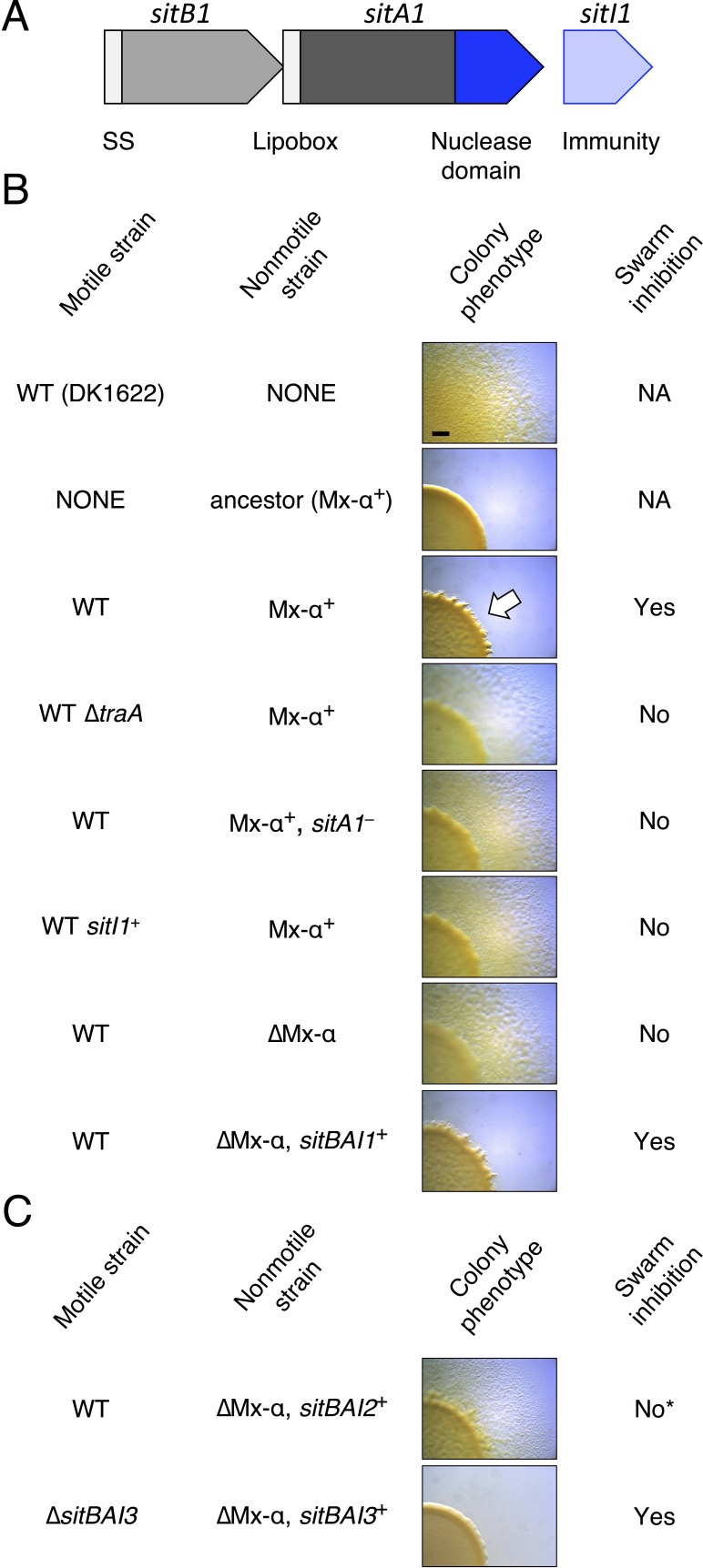


The article has been corrected accordingly.

