# Supplementary material for: Infectious polymorphic toxins delivered by outer membrane exchange discriminate kin in myxobacteria
Source: eLife. 2017 Aug 18;6:e29397. doi: 10.7554/eLife.29397 (PMC5562445; doi:10.7554/eLife.29397)
Supplement: Supplementary file 1. — DOI: http://dx.doi.org/10.7554/eLife.29397.016 [file elife-29397-supp1.docx]

Supplementary file 1A Homologs of SitA1 N-terminus^a^

| Organism | Locus Tag | Positives | e value | Annotated length (aa) | SitB Upstream (Y/N) | -3 to +7 Lipobox |
| --- | --- | --- | --- | --- | --- | --- |
| *Myxococcus xanthus* DK101 | | | | | | |
|  | MXF1DRAFT_07513 (SitA1) | 507/507 (100%) | 0 | 587 | yes | ASGCAALPARP |
|  | MXF1DRAFT_07313 (SitA2) | 314/436 (72%) | 0 | 784 | yes | ASGCASLPARP |
| *Myxococcus virescens* | | | | | | |
|  | Ga0070493_13031 | 373/432 (86%) | 0 | 777 | yes | ASGCAVLPARP |
| *Hyalangium minutum* | | | | | | |
|  | Ga0055551_103952 | 252/354 (71%) | 1E-103 | 699 | no | WSGCVPVHGSA |
|  | Ga0055551_111185 | 257/350 (73%) | 7E-98 | 698 | no | VSGCTPVQSNA |
|  | Ga0055551_11867 | 181/250 (72%) | 2E-84 | 353 | no | VSGCVPVHGTA |
| *Stigmatella erecta* | | | | | | |
|  | Ga0131206_1335 | 264/384 (69%) | 7E-105 | 731 | yes | TAGCASLPSRA |
| *Cystobacter violaceus* | | | | | | |
|  | Q664_24205 | 293/452 (65%) | 2E-121 | 749 | no | LAGCGTGHPRG |
|  | Q664_38065 | 239/325 (74%) | 2E-113 | 662 | unknown | End of contig |
|  | Q664_14010 | 271/377 (72%) | 3E-113 | 756 | no | TTACATRAPHS |
|  | Q664_46200 | 215/329 (65%) | 3E-93 | 765 | no | STGCVTLAPRQ |
|  | Q664_08725 | 209/329 (64%) | 4E-86 | 688 | no | STGCVTLAPRQ |
|  | Q664_52095 | 223/336 (66%) | 2E-79 | 588 | no | LGGCTTAGASV |
|  | Q664_19980 | 197/329 (60%) | 3E-67 | 735 | no | FTGCVTLAPRQ |
|  | Q664_18030 | 196/329 (60%) | 5E-64 | 778 | no | STGCVTLAPRQ |
| *Stigmatella aurantiaca* DW4/3-1 | | | | | | |
|  | STAUR_2670 | 365/416 (88%) | 0 | 721 | yes | ASGCASLPARP |
|  | STAUR_2564 | 352/422 (83%) | 0 | 723 | yes | ASGCASLPARP |
|  | STAUR_3157 | 311/384 (81%) | 2E-159 | 729 | yes | AMGCGTLTSRA |
|  | STAUR_2461 | 255/360 (71%) | 7E-102 | 720 | yes | TVGCASLPSRA |
| *Myxococcus* sp. | | | | | | |
|  | A176_04204 | 313/354 (88%) | 4E-161 | 656 | unknown | End of contig |
| *Cystobacter fuscus* | | | | | | |
|  | D187_007419 | 249/362 (69%) | 2E-110 | 560 | no | LAGCATSAPLG |
|  | D187_010442 | 255/379 (67%) | 1E-102 | 645 | no | LTGCATSAPLA |
|  | D187_004722 | 236/325 (73%) | 4E-98 | 716 | no | LAGCAASTALV |
|  | D187_002100 | 249/387 (64%) | 8E-93 | 729 | no | GRGCAMVARRV |
|  | D187_007405 | 204/322 (63%) | 5E-80 | 661 | no | LTGCATGRPHE |
|  | D187_006214 | 163/254 (64%) | 9E-69 | 340 | no | LAGCATGAIGG |
|  | D187_006857 | 205/329 (62%) | 3E-66 | 757 | no | STGCVTLAPRQ |
|  | D187_006659 | 140/203 (69%) | 2E-42 | 487 | unknown | End of contig |
| *Myxococcus fulvus* HW-1 | | | | | | |
|  | LILAB_17325 | 364/433 (84%) | 0 | 783 | yes | ASGCASLPARP |
|  | LILAB_05795 | 231/323 (72%) | 1E-103 | 597 | yes | SSGCASLPSRT |
| *Archangium gephyra* | | | | | | |
|  | Ga0081691_11931 | 276/395 (70%) | 1E-121 | 750 | no | LAGCATDHPRG |
|  | Ga0081691_115975 | 277/412 (67%) | 2E-107 | 683 | no | WCGCVTDRPHR |
|  | Ga0081691_112377 | 215/330 (65%) | 3E-94 | 679 | no | QGGCTTEAVDT |
|  | Ga0081691_115086 | 210/321 (65%) | 1E-83 | 754 | no | STGCVTLTPRQ |
| *Myxococcus fulvus* | | | | | | |
|  | Ga0131203_101593 | 297/376 (79%) | 2E-157 | 621 | yes | NVGCASLPSKS |
|  | Ga0131203_101910 | 298/378 (79%) | 2E-156 | 628 | yes | NVGCASLPAKS |
|  | Ga0131203_102957 | 226/324 (70%) | 8E-80 | 587 | yes | TVGCASRAGSD |
| *Corallococcus coralloides* | | | | | | |
|  | COCOR_07554 | 328/401 (82%) | 2E-164 | 700 | yes | NAGCASLSARQ |
|  | COCOR_05731 | 260/317 (82%) | 7E-126 | 568 | no | IAGCASSSSHG |
|  | COCOR_07546 | 279/416 (67%) | 4E-109 | 733 | yes | TAGCASLPSRA |
|  | COCOR_01926 | 259/360 (72%) | 8E-108 | 687 | yes | TSGCASLPSRA |

^a^Homologs of SitA1/SitA2 conserved region (residues 1-507 of SitA1) from the IMG database (Markowitz et al., 2012) are found only within the order Myxococcales. Each homolog has a lipobox with conserved glycine or alanine followed by an invariant cysteine (red text). The absence of a lysine at the +2 position suggests these protein are sorted to the OM (Bhat et al., 2011). Highlighted gene was heterologously expressed in DK1622 (Fig. 1 – figure supplement 3).

Supplementary file 1B Homologs of SitA3 N-terminus^a^

| Organism | Locus Tag | Positives | e value | Annotated length | SitB Upstream (Y/N) | -3 to +7 Lipobox |
| --- | --- | --- | --- | --- | --- | --- |
| *Myxococcus xanthus* DK1622/DK101 | | | | | | |
|  | MXAN_1899 (SitA3) | 441/441 (100%) | 0 | 543 | yes | ASGCATLTPLS |
| *Myxococcus virescens* | | | | | | |
|  | Ga0070493_10971 | 444/452 (98%) | 0 | 543 | yes | ASGCATLTPLS |
| *Hyalangium minutum* | | | | | | |
|  | Ga0055551_11780 | 149/272 (55%) | 8E-41 | 467 | no | LTGCASSTSAS |
| *Stigmatella erecta* | | | | | | |
|  | Ga0131206_109286 | 151/272 (56%) | 3E-47 | 464 | no | LSGCATGHTTG |
| *Stigmatella aurantiaca* | | | | | | |
|  | STAUR_3680 | 73/121 (60%) | 3E-22 | 155 | no | IS element insertion |
| *Cystobacter violaceus* | | | | | | |
|  | Q664_23910 | 247/372 (66%) | 2E-122 | 506 | no | STGCITVMPPS |
|  | Q664_37715 | 154/282 (55%) | 5E-34 | 570 | no | QTACATGYPMG |
|  | Q664_46455 | 142/278 (51%) | 1E-33 | 607 | no | QTACATGYPMG |
|  | Q664_26250 | 148/279 (53%) | 7E-29 | 535 | no | LTACATGHPMS |
|  | Q664_19210 | 130/237 (55%) | 2E-28 | 579 | no | QAACATGSPMG |
|  | Q664_23930 | 126/237 (53%) | 7E-28 | 605 | no | QTACATGSPMG |
|  | Q664_32810 | 51/85 (60%) | 8E-15 | 117 | no | Truncated |
|  | Q664_19545 | 61/104 (59%) | 4E-12 | 249 | no | LQGCATGNPRG |
|  | Q664_45905 | 70/149 (47%) | 9E-10 | 191 | no | ATACASRPPPE |
| *Myxococcus* sp. | | | | | | |
|  | A176_07430 | 248/364 (68%) | 2E-103 | 536 | no | ATGCASTSPAS |
|  | A176_03028 | 108/140 (77%) | 3E-44 | 299 | no | LQGCATGNPRG |
|  | A176_03025 | 89/129 (69%) | 6E-39 | 173 | no | Truncated |
|  | A176_06363 | 35/54 (65%) | 3E-11 | 63 | no | Truncated |
| *Cystobacter fuscus* | | | | | | |
|  | D187_002886 | 260/379 (69%) | 3E-128 | 523 | no | STGCITVTPPV |
|  | D187_005663 | 247/355 (70%) | 2E-127 | 498 | yes | STGCITVTPSS |
|  | D187_005679 | 251/363 (69%) | 2E-126 | 479 | no | STGCITVTPSS |
|  | D187_002899 | 250/365 (68%) | 1E-124 | 485 | no | FTGCVTVTPPA |
|  | D187_007265 | 210/281 (75%) | 7E-114 | 429 | no | ATGCATVTTAS |
|  | D187_001070 | 151/267 (57%) | 1E-39 | 502 | no | LTGCVTGAPRG |
|  | D187_005785 | 150/274 (55%) | 4E-39 | 511 | no | IQGCASVRHNA |
|  | D187_002296 | 150/273 (55%) | 1E-37 | 458 | no | LEGCATGHPRG |
|  | D187_001068 | 62/100 (62%) | 2E-13 | 275 | no | Truncated |
|  | D187_006246 | 71/153 (46%) | 4E-10 | 173 | no | ATACASRPPPE |
| *Myxococcus fulvus* HW-1 | | | | | | |
|  | LILAB_13250 | 363/378 (96%) | 0 | 539 | yes | ASGCATLTPLS |
|  | LILAB_02555 | 202/273 (74%) | 1E-97 | 438 | no | IS element insertion |
|  | LILAB_02580 | 200/273 (73%) | 4E-94 | 511 | no | LTGCAGVESST |
|  | LILAB_14775 | 191/270 (71%) | 9E-92 | 498 | no | FMGCSGVESST |
| *Archangium gephyra* | | | | | | |
|  | Ga0081691_118545 | 146/271 (54%) | 1E-36 | 523 | no | LGGCATAPPR |
|  | Ga0081691_111205 | 143/279 (51%) | 2E-36 | 588 | no | LGGCATSAPLP |
|  | Ga0081691_111641 | 89/170 (52%) | 9E-20 | 285 | no | LTGCTTGAHWR |
|  | Ga0081691_11276 | 57/119 (48%) | 1E-9 | 366 | no | LTGCTTGAHWR |
| *Myxococcus fulvus* | | | | | | |
|  | Ga0131203_104307 | 199/272 (73%) | 5E-101 | 496 | no | LTGCAGVESSA |
|  | Ga0131203_110142 | 193/272 (71%) | 1E-91 | 498 | no | VTGCAGGDASS |
| *Myxococcus stipitatus* | | | | | | |
|  | MYSTI_04959 | 197/272 (72%) | 7E-94 | 465 | no | ATGCAGVDASA |
|  | MYSTI_03282 | 198/272 (73%) | 2E-93 | 524 | no | ATGCAGVDAST |
| *Corallococcus coralloides* | | | | | | |
|  | COCOR_00885 | 147/202 (73%) | 6E-74 | 454 | no | LVGCAGGRARA |

^a^Homologs of SitA3 conserved region (residues 1-441) from the IMG database (Markowitz et al., 2012) are found only within the order Myxococcales. Each homolog has a lipobox with conserved glycine or alanine followed by an invariant cysteine (red text). The absence of a lysine at the +2 position suggests these proteins are sorted to the OM (Bhat et al., 2011). Highlighted gene was heterologously expressed in DK1622 (Fig. 1 – figure supplement 3).
