# Supplementary material for: Infectious polymorphic toxins delivered by outer membrane exchange discriminate kin in myxobacteria
Source: eLife. 2017 Aug 18;6:e29397. doi: 10.7554/eLife.29397 (PMC5562445; doi:10.7554/eLife.29397)
Supplement: Supplementary file 2. — DOI: http://dx.doi.org/10.7554/eLife.29397.017 [file elife-29397-supp2.docx]

Supplementary file 2A Strains used in this study

| Strain | Genotype | General description/Experimental use | Source |
| --- | --- | --- | --- |
| DK1622 | Reference *M. xanthus* | WT ∆Mx-α for repeats 1-2 (Figs. 1B, 1C, 2B) | Wall et al., 1999; Dey et al., 2016 |
| MG1655 | Reference *E. coli* | WT (parent strain) | ATCC |
| TOP10 | *E. coli* cloning strain | Cloning | Invitrogen |
| DW709 | DK1622 ∆*pilA* (markerless) *gfp* (inserted at Mx8 *att* site, Km^r^) | Non-clumping, Km^r^ target strain for CFU count (Fig. 5A) | Wei et al., 2014 |
| DW1480 | DK1622 ∆*traA* | WT ∆*traA* (Figs. 1B, 2B) | Vassallo et al., 2015 |
| DK8601^a^ | ∆*pilA aglB1* (*aglQ1*) | Nonmotile ancestor (Fig 1B) | Wall et al., 1998 |
| DK6204 | DK1622 ∆*mglBA* | Nonmotile ∆Mx-α for repeats 1-2 (Fig. 1B) | Hartzell and Kaiser, 1991 |
| DW2408^a^ | DK8601 *sitA1*::pCR TOPO 2.1 | Nonmotile ancestor *sitA1*^–^ (Fig. 1B) | This study |
| DW2409 | DK1622 P_IPTG_-*sitI1* | WT *sitI1*^+^ (Fig. 1B) | This study |
| DW2410 | DK6204 P_IPTG_-*sitBAI1* | Nonmotile ∆Mx-α *sitBAI1*^+^ (Fig. 1B) | This study |
| DW2411 | DK6204 P_IPTG_-*sitBAI2* | Nonmotile ∆Mx-α *sitBAI2*^+^ (Fig. 1C) | This study |
| DW2412 | DK6204 P_IPTG_-*sitBAI3* | Nonmotile ∆Mx-α *sitBAI3*^+^ (Fig. 1C) | This study |
| DW2413 | DK1622 P_IPTG_-*tdTomato* | Target for *sitBAI1*^+^ or *sitBAI2*^+^ (Figs. 1B, 7A, 7B, 2S3) | This study |
| DW2414 | DK1622 ∆*traA* P_IPTG_-*tdTomato* | ∆*traA* target (Figs. 1B, 2B,7A, 7B, 2S3) | This study |
| DW2415 | DK1622 P_IPTG_-*sitBAI1* | *sitBAI1*^+^ (Figs. 2B, 2D, 4B, 5A) | This study |
| DW2416 | DK1622 P_IPTG_-*sitBAI2* | *sitBAI2*^+^ (Figs. 2B & 4B) | This study |
| DW2417 | DK1622 P_IPTG_-*sitBAI3* | *sitBAI3*^+^ (Fig. 2B) | This study |
| DW2418 | DK1622 ∆*traA* P_IPTG_-*sitBAI1* | ∆*traA* *sitBAI1*^+^ (Fig. 2B) | This study |
| DW2419 | DK1622 ∆*traA* P_IPTG_-*sitBAI2* | ∆*traA sitBAI2*^+^ (Figs. 2B & 4B) | This study |
| DW2420 | DK1622 ∆*traA* P_IPTG_-*sitBAI3* | ∆*traA sitBAI3*^+^ (Fig. 2B) | This study |
| DW2421 | DK1622 *sitAI3*::pCR TOPO XL P_IPTG_-*tdTomato* | *sitAI3*^–^, target for *sitBAI3*^+^ (Fig. 2B) | This study |
| DW2422 | DK1622 ∆*traA*, *sitAI3*::pCR TOPO XL P_IPTG_-*tdTomato* | ∆*traA* *sitAI3*^–^, target for *sitBAI3*^+^ (Fig. 2B) | This study |
| DW2423 | DK1622 *∆sitBAI3* P_IPTG_-*sitBAI1* P_R3,4_-*egfp* | *sitIBA1*^+^ GFP (Fig. 2E) | This study |
| DW2424 | DK1622 ∆*sitBAI3* P_IPTG_-*sitBAI2* P_R3,4_-*egfp* | *sitIBA2*^+^ GFP (Fig. 2E) | This study |
| DW2425 | DK1622 ∆*sitBAI3* P_IPTG_-*sitBAI3* P_R3,4_-*egfp* | *sitIBA3*^+^ GFP (Fig. 2E) | This study |
| DW2426 | DK1622 ∆*sitBAI3* P_IPTG_-*sitI1* | *sitI1*^+^ (Fig. 2E) | This study |
| DW2427 | DK1622 ∆*sitBAI3* P_IPTG_-*sitI2* | *sitI2*^+^ (Fig. 2E) | This study |
| DW2428 | DK1622 ∆*sitBAI3* P_IPTG_-*sitI3* | *sitI3*^+^ (Fig. 2E) | This study |
| DW2429 | DK1622 P_IPTG_-*sitA1*-*mCherry* | SitA1 lipobox mCherry fusion reporter (Fig. 2C) | This study |
| DW2430 | DK1622 ∆*traA* P_R3,4_-*egfp* | ∆*traA* reporter target (Fig. 2C) | This study |
| DW2431 | DK1622 P_IPTG_-*sitBA_FLAG_I1* | C-terminal SitA1-FLAG fusion (Fig. 2D) | This study |
| DW2432 | DK1622 ∆*sitBAI3* P_IPTG_-*sitA1*-*CTD* | Inducible SitA1-CTD toxin domain (Fig. 2S2) | This study |
| DW2433 | DK1622 ∆*sitBAI3* P_IPTG_-*sitA2*-*CTD* | Inducible SitA2-CTD toxin domain (Fig. 2S2) | This study |
| DW2434 | DK1622 ∆*sitBAI3* P_IPTG_-*sitA3*-*CTD* | Inducible SitA3-CTD toxin domain (Fig. 2S2) | This study |
| DW2435 | DK1622 ∆*sitBAI3* | ∆*sitBAI1*-*3* (Figs. 1C, 4A, 7A, 7B, 2S1) | This study |
| DW2436 | DK1622 ∆*sitBAI3*, P_IPTG_-*sitBAI1* | *sitBAI1*^+^ (Figs. 4A, 6A, S1) | This study |
| DW2437 | DK1622 ∆*sitBAI3* P_IPTG_-*sitBAI2* | *sitBAI2*^+^ (Figs. 4A & 2S1) | This study |
| DW2438 | DK1622 ∆*sitBAI3* P_IPTG_-*sitBAI3* | *sitBAI3*^+^ (Figs. 4A & 2S1) | This study |
| DW2439 | DK1622 ∆*sitBAI3* P_IPTG_-*sitAI3^Mf1^* | *sitAI3^Mf1^*^+^ (Figs. 4A) | This study |
| DW2440 | DK1622 ∆*sitBAI3* P_IPTG_-*sitBAI1^Mf1^* | *sitBAI1^Mf1^*^+^ (Figs. 4A) | This study |
| DW2441 | DK1622 P_IPTG_-*sitAI3^Mf1^* | *sitAI3^Mf1^*^+^ (Fig. 2S3) | This study |
| DW2442 | DK1622 P_IPTG_-*sitBAI1^Mf1^* | *sitBAI1^Mf1^*^+^ (Fig. 2S3) | This study |
| DW2443 | DK1622 ∆*traA* P_IPTG_-*sitBAI1*, P*_pilA_*-*traAB^Mf^* | Toxin producer *traAB^Mf^* for infection exp. (Figs. 5C-H, 6B) | This study |
| DW2444 | DK1622 P_IPTG_-*tdTomato* P*_pilA_*-*traAB^Mf^* | *traA* diploid for infection exp. (Figs. 5D-I, 6B) | This study |
| DW2445 | DK1622 P_R3,4_-*egfp* | GFP target (Figs. 2C, 5C, 5E-I, 6B) | This study |
| DW2446 | DK1622 P_R3,4_-*egfp* P_IPTG_-*sitI1* | Target strain for infection exp., *sitI1*^+^ (Fig. 5G) | This study |
| DW2447 | DK1622 ∆*sitBAI3* P_IPTG_-*tdTomato* | General target strain (∆*sitBAI1*-*3*) (Figs. 6A, 7A, 7B, 2S1, 2S2) | This study |
| DW2448 | DK1622 ∆*traA* P*_pilA_*-*traAB^Mf^* P_IPTG_-*sitA1*-*mCherry* | SitA1-mCherry fusion reporter for infection exp. (Fig. 5I) | This study |
| DW2249 | DK1622 ∆*sitBAI3* P_IPTG_-*sitAI1* | ∆SitB3 SitAI inhibitor (∆SitB) (Fig. 6A) | This study |
| DW2250 | DK1622 ∆*traA* P*_pilA_*-*traAB^Mf^* P_IPTG_-*sitAI1* | SitAI inhibitor for infection exp. (Fig. 6B) | This study |
| DW2251^a^ | DK8601 P*_pilA_*-SS_OM_-*sfgfp* | sfGFP reporter for TraA transfer exp. (Fig. 5S1) | This study |
| DW2252^a^ | DK8601 ∆*traAB* P*_pilA_*-SS_OM_-*sfgfp* | ∆*traAB* sfGFP reporter for TraA transfer exp. (Fig. 5S1) | This study |
| DW2253 | DK1622 ∆*pilA* ∆*traAB* P*_pilA_*-*traA*-*mCherry*-*traB* | TraA-mCherry reporter for TraA transfer exp. (Fig. 5S1) | This study |
| DW1414 | DK1622 ∆*pilQ* P*_pilA_*-*gfp* | Reporter transfer target for TraA transfer exp. (Fig. 5S1) | Pathak et al., 2012 |
| DW1484 | DK1622 ∆*pilQ* P*_pilA_*-SS_OM_-*mCherry* | Control reporter for TraA transfer exp. (Fig. 5S1) | This study |
| A66 | Environmental *M. xanthus* isolate | DK1622 *traA* recognition group (Fig. 7A) | Vos and Velicer, 2006 |
| A88 | Environmental *M. xanthus* isolate | DK1622 *traA* recognition group (Fig. 7A) | Vos and Velicer, 2006 |
| DK801 | Environmental *M. xanthus* isolate | DK1622 *traA* recognition group (Fig. 7A) | Martin et al., 1978 |
| A23 | Environmental *M. xanthus* isolate | Outside DK1622 *traA* recognition group (Fig. 7B) | Vos and Velicer, 2006 |
| A47 | Environmental *M. xanthus* isolate | Outside DK1622 *traA* recognition group (Fig. 7B) | Vos and Velicer, 2006 |
| CV192 | MG1655 empty pBAD30 empty pKSAT | *E. coli* empty vector control (Fig. 2F) | This study |
| CV178 | MG1655 pBAD30-*sitA1*-CTD empty pKSAT | *E. coli* *sitA1*-CTD (Fig. 2F) | This study |
| CV143 | MG1655 pBAD30-*sitA1*-CTD pKSAT-*sitI1* | *E. coli* *sitA1*-CTD *sitI1* (Fig. 2F) | This study |
| CV144 | MG1655 pBAD30-*sitA1*-CTD pKSAT-*sitI2* | *E. coli* *sitA1*-CTD *sitI2* (Fig. 2F) | This study |
| CV167 | MG1655 pBAD30-*sitA1*-CTD pKSAT-*sitI3* | *E. coli* *sitA1*-CTD *sitI3* (Fig. 2F) | This study |
| CV180 | MG1655 pBAD30-*sitA3*-CTD empty pKSAT | *E. coli* *sitA3*-CTD (Fig. 2F) | This study |
| CV169 | MG1655 pBAD30-*sitA3*-CTD pKSAT-*sitI1* | *E. coli* *sitA3*-CTD *sitI1* (Fig. 2F) | This study |
| CV171 | MG1655 pBAD30-*sitA3*-CTD pKSAT-*sitI2* | *E. coli* *sitA3*-CTD *sitI2* (Fig. 2F) | This study |
| CV165 | MG1655 pBAD30-*sitA3*-CTD pKSAT-*sitI3* | *E. coli* *sitA3*-CTD *sitI3* (Fig. 2F) | This study |
| CV125 | MG1655 pBAD30-*sitA1*-CTD | *E. coli* *sitA1*-CTD DAPI exp. (Fig. 3B) | This study |
| CV159 | MG1655 pBAD30-*sitA3*-CTD | *E. coli* *sitA1*-CTD DAPI exp. (Fig. 3B) | This study |
| CdiA-CT^Bp^ | MG1655 pCH450-*cdiA*-CTD^Bp1026b^ | Northern blot exp. (Fig. 3A) | Nikolakakis et al., 2012 |
| CdiA-CT^Yp^ | MG1655 pCH450-*cdiA*-CTD^YPIII^ | Northern blot exp. (Fig. 3A) | This study |
| SitA3-CT | MG1655 pCH450-*sitA3*-CTD | Northern blot exp. (Fig. 3A) | This study |

^a^Derived from DK101 ancestor strain (contains all three Mx-alpha repeats)

Supplementary file 2B Plasmids used in this study

| Plasmid | Relevant properties | Source |
| --- | --- | --- |
| pCR 2.1 TOPO | Cloning vector, Km^r^ | Invitrogen |
| pMR3487 | IPTG-inducible promoter, Tc^r^ | Iniesta et al., 2012 |
| pBAD30 | Arabinose inducible promoter, Amp^r^ | Guzman et al., 1995 |
| pKSAT | Constitutive promoter, Sm^r^ | Sumiko Inouye |
| pSWU19 | *M. xanthus* cloning vector, Km^r^ | Wu and Kaiser, 1995 |
| pTdTomato | pMR3487-*tdTomato*, Tc^r^ | Larry Shimkets |
| pXW6 | P*_pilA_*-SS_OM_-*mCherry* in pKSAT | Wei et al., 2011 |
| pBJ114 | Deletion cassette plasmid, Km^r^-Gal^s^ | Julien et al., 2000 |
| pCV4 | *sitA1* fragment in pCR TOPO 2.1, Km^r^ | This study |
| pCV5 | pMR3487-*sitI1*, Tc^r^ | This study |
| pCV6 | pMR3487-*sitBAI1*, Tc^r^ | This study |
| pCV7 | pMR3487-*sitBAI2*, Tc^r^ | This study |
| pCV8 | pMR3487-*sitBAI3*, Tc^r^ | This study |
| pCV9 | *sitA3* fragment in pCR TOPO XL, Km^r^ | This study |
| pCV10 | pSWU19-P_R3,4_-*egfp*, Km^r^ | This study |
| pCV11 | pMR3487-*sitI2*, Tc^r^ | This study |
| pCV12 | pMR3487-*sitI3*, Tc^r^ | This study |
| pCV13 | pMR3487-*sitAI3^Mf1^*, Tc^r^ | This study |
| pCV14 | pMR3487-*sitBAI1^Mf2^*^,^ Tc^r^ | This study |
| pCV16 | pBAD30-*sitA1*-CTD, Ap^r^ | This study |
| pCV17 | pBAD30-*sitA3*-CTD, Ap^r^ | This study |
| pCV18 | pKSAT-*sitI1*, Sm^r^ | This study |
| pCV19 | pKSAT-*sitI2*, Sm^r^ | This study |
| pCV20 | pKSAT-*sitI3*, Sm^r^ | This study |
| pCV21 | *sitBAI3* deletion cassette in pBJ114, Km^r^-Gal^s^ | This study |
| pCV22 | pMR3487-*sitA1*-mCherry, Tc^r^ | This study |
| pCV23 | pMR3487-*sitBA_FLAG_I1*, Tc^r^ | This study |
| pCV24 | pMR3487-*sitAI1*, Tc^r^ | This study |
| pCV25 | pMR3487-*sitA1*-CTD, Tc^r^ | This study |
| pCV26 | pMR3487-*sitA2*-CTD, Tc^r^ | This study |
| pCV27 | pMR3487-*sitA3*-CTD, Tc^r^ | This study |
| pPC1 | P*_pilA_*-SS_OM_-*sfgfp*in pKSAT | Vassallo and Wall, 2016 |
| pPC4 | pSWU19-P*_pilA_*-*traAB^M^*^.^ *^fulvus^*, Km^r^ | Cao and Wall, 2017 |
| pPC25 | P*_pilA_*-*traA*-*mCherry*-*traB*in pSWU19, Km^r^ | This study |
| pCH450 | pACYC184-derivative with arabinose inducible promoter, Tc^r^ | Hayes and Sauer, 2003 |
| pCH10130 | pCH450-*cdiA*-*CT*^Bp1026b^, Tc^r^ | Nikolakakis et al., 2012 |
| pCH10648 | pCH450-YPK_0573-CT, Tc^r^ | This study |
| pCH12419 | pCH450-*sitA3*-*CT*, Tc^r^ | This study |

Supplementary file 2C Primers used in this study^a^

| Primer Name | Sequence (5’-3’) |
| --- | --- |
| TOPO*sitA1*insertion-F | TTGGTGGTGGCTCTCCTG |
| TOPO*sitA1*insertion-R | CGGACGACAGGCATGCTG |
| KpnI-*sitI1*-F | GACGACTCTAGAATGGAGCATCTTGTGCAGACG |
| XbaI-*sitI1*-R | GACGACGGTACCCTACACCTTGAGAACGCTAGGG |
| Gibson-*sitBAI1*-F | TGAGCGGATAACAATTAAGGAGGCTCTAGAATGTTCATGCGCTGGGCC |
| Gibson-*sitBAI1*-R | ATGATTACGAAGGCGAGCTCGGTACCCTACACCTTGAGAACGCTAGGGG |
| Gibson-*sitBAI2*-F | TGAGCGGATAACAATTAAGGAGGCTCTAGAATGTTCACGCGCTGGGCC |
| Gibson-*sitBAI2*-R | ATGATTACGAAGGCGAGCTCGGTACCTCAGGGCCGGTACCGCTC |
| XbaI-*sitBAI3*-F | GACGACTCTAGAATGTCTCTGAGTCGATGTTGG |
| KpnI-*sitBAI3*-R | GACGACGGTACCTCAGAGCAATGGCGAATTTAG |
| TOPO*sitA3*insertion-F | GCTGAGTCTCTGGGGAAGTG |
| TOPO*sitA3*insertion-R | CATCAAGGAGGCGGAAGAT |
| Gibson-*egfp*-F | CAGAAGAAGACGGTAGCAATAGTAGATAAGGAGGAGGTTAGAAATGGTGAGCAAGGGCGAG |
| Gibson-*egfp*-R | GTCACGACGTTGTAAAACGACGCCAAGCTTTTACTTGTACAGCTCGTCCATG |
| Gibson-PR34promoter-F | GGAAACAGCTATGACCATGATTACGAATTCGACGAGTCGAGGGAGTCAAC |
| Gibson-PR34promoter-R | TCCTTATCTACTATTGCTACCGTCTTCTTCTGCCCGG |
| XbaI-*sitI2*-F | GACGACTCTAGAATGAAGGCGCTGACACTGCG |
| KpnI-*sitI2*-R | GACGACGGTACCTCAGGGCCGGTACCGCTC |
| XbaI-*sitI3*-F | GACGACTCTAGAATGAAGGCGCTGACACTGCG |
| KpnI-*sitI3*-R | GACGACGGTACCTCAGGGCCGGTACCGCTC |
| XbaI-*sitAIMF1*-F | GACGACTCTAGAATGCGTGCTGGCCATTGG |
| KpnI-*sitAIMF1*-R | GACGACGGTACCCTATGGTTGGGCCTGGAGCG |
| Gibson-*sitBAIMF2*-F | CTAGATGAGCGGATAACAATTAAGGAGGCTCTAGAATGTTTGTGTGCTGGAGTTGG |
| Gibson-*sitBAIMF2*-R | CATGATTACGAAGGCGAGCTCGGTACCCCGACAAGGCGATGAAGC |
| EcoRI-*sitA1*-CT-F | GACGACGAATTCATGGACGTTCCCATTCGGAATGC |
| HindIII-*sitA1*-CT-R | GACGACAAGCTTATGTTATTGGCCTCCTGCGAAG |
| EcoRI-*sitA3*-CT-F | GACGACGAATTCATGGGGAGCCTTACAGGACGA |
| HindIII-*sitA3*-CT-R | GACGACAAGCTTTCAAGGGTAAATCAAGGAGACC |
| NdeI-*sitI1*-F | GACGACCATATGGAGCATCTTGTGCAGACGG |
| BamHI-*sitI1*-R | GACGACGGATCCCTACACCTTGAGAACGCTAGG |
| NdeI-*sitI2*-F | GACGACCATATGAAGGCGCTGACACTGCGTG |
| BamHI-*sitI2*-R | GACGACGGATCCTCAGGGCCGGTACCGCTC |
| NdeI-*sitI3*-F | GACGACCATATGGGTCTTGAGAATGGGCTT |
| BamHI-*sitI3*-R | GACGACGGATCCTCAGAGCAATGGCGAATT |
| Gibson-∆*sitBAI3*-Upstream-F | AACACGTATGACCATGATTACGCCAAGCTTCCACTGTGCTGCGTACATG |
| Gibson-∆*sitBAI3*-Upstream-R | GGATTTTGTAGGGCATCCCGGGCAATAACAA |
| Gibson-∆*sitBAI3*-Downstream-F | ATTGCCCGGGATGCCCTACAAAATCCAGCCC |
| Gibson-∆*sitBAI3*-Downstream-R | CGACGTTGTAAAACGACGGCCAGTGAATTCACTCATGAGCTGACCACC |
| Gibson-*sitA1*-*mCherry*-Upstream-F | TGAGCGGATAACAATTAAGGAGGCTCTAGATGCGCGCTGAGCGGCGT |
| Gibson-*sitA1*-*mCherry*-Upstream-R | CGGAGGAGGCCATCCTCGCGGGCAGTGCAGC |
| Gibson-*sitA1*-*mCherry*-Downstream-F | ACTGCCCGCGAGGATGGCCTCCTCCGAGGAC |
| Gibson-*sitA1*-*mCherry*-Downstream-R | ATGATTACGAAGGCGAGCTCGGTACCTTACTTGTACAGCTCGTCCATG |
| Gibson-*sitBA_FLAG_I*-Upstream-F | TGAGCGGATAACAATTAAGGAGGCTCTAGAATGTTCATGCGCTGGGCC |
| Gibson-*sitBA_FLAG_I*-Upstream-R | CGTCGTCCTTGTAGTCTTGGCCTCCTGCGAAGCC |
| Gibson-*sitBA_FLAG_I*-Downstream-F | CGCAGGAGGCCAAGACTACAAGGACGACGACGACAAGTAATGGAGCATCTTGTGCAGAC |
| Gibson-*sitBA_FLAG_I*-Downstream-R | ATGATTACGAAGGCGAGCTCGGTACCCTACACCTTGAGAACGCTA G |
| XbaI-*sitAI*-F | GACGACTCTAGAATGCGCGCTGAGCGGCGT |
| KpnI-*sitAI*-R | GACGACGGTACCCCGGTCGCCGCACCAGCA |
| XbaI-*sitA1*-*CTD*-F | GACGACTCTAGAATGGACGTTCCCATTCGGAATGC |
| KpnI-*sitA1*-*CTD*-R | GACGACGGTACCTTATTGGCCTCCTGCGAAG |
| XbaI-*sitA2*-*CTD*-F | GACGACTCTAGAATGGCAGGGGCGCGGTACGT |
| KpnI-*sitA2*-*CTD*-R | GACGACGGTACCTCATTGAAACACCACCTCCACGCC |
| XbaI-*sitA3*-*CTD*-F | GACGACTCTAGAATGAAGCCATGGTGCCCATCC |
| KpnI-*sitA3*-*CTD*-R | GACGACGGTACCTCAAGGGTAAATCAAGGAGACC |
| NcoI-*cdiA*-*CT*-YP-F | ACACCATGGCCAAATCAGACGGTTTATTGAC |
| XhoI-*cdiA*-*CT*-YP-R | GGACTCGAGTCCTTACTTCACGCG |
| NcoI-*sitA3*-*CT*-F | AAGCCATGGTGCCCATCCTCTCTCTCTC |
| XhoI-*sitA3*-*CT*-R | AACCTCGAGCACCTGCTTCAAG |

^a^F, forward. R, reverse.

References

Cao P, Wall D. 2017. Self-identity reprogrammed by a single residue switch in a cell surface receptor of a social bacterium. *PNAS* **114**:3732–3737. **PubMed:** [28320967](http://www.ncbi.nlm.nih.gov/pubmed/28320967) **DOI:** [10.1073/pnas.1700315114](http://dx.doi.org/10.1073/pnas.1700315114)

Dey A, Vassallo CN, Conklin AC, Pathak DT, Troselj V, Wall D. 2016. Sibling rivalry in Myxococcus xanthus is mediated by kin recognition and a polyploid prophage. Journal of Bacteriology **198**:994–1004. **PubMed:** [26787762](http://www.ncbi.nlm.nih.gov/pubmed/26787762) **DOI:** [10.1128/JB.00964-15](http://dx.doi.org/10.1128/JB.00964-15)

Guzman LM, Belin D, Carson MJ, Beckwith J. 1995. Tight regulation, modulation, and high-level expression by vectors containing the arabinose PBAD promoter. *Journal of Bacteriology* **177**:4121–4130. **PubMed:** [7608087](http://www.ncbi.nlm.nih.gov/pubmed/7608087)**DOI:** [10.1128/jb.177.14.4121-4130.1995](http://dx.doi.org/10.1128/jb.177.14.4121-4130.1995)

Hartzell P, Kaiser D. 1991. Upstream gene of the *mgl* operon controls the level of MglA protein in *Myxococcus xanthus*. *Journal of Bacteriology* **173**:7625–7635. **PubMed:** [1938958](http://www.ncbi.nlm.nih.gov/pubmed/1938958) **DOI:** [10.1128/jb.173.23.7625-7635.1991](http://dx.doi.org/10.1128/jb.173.23.7625-7635.1991)

Hayes CS, Sauer RT. 2003. Cleavage of the A site mRNA Codon during ribosome pausing provides a mechanism for translational quality control. *Molecular Cell* **12**:903–911. **PubMed:** [14580341](http://www.ncbi.nlm.nih.gov/pubmed/14580341) **DOI:** [10.1016/S1097-2765(03)00385-X](http://dx.doi.org/10.1016/S1097-2765(03)00385-X)

Iniesta AA, García-Heras F, Abellón-Ruiz J, Gallego-García A, Elías-Arnanz M. 2012. Two systems for conditional gene expression in *Myxococcus xanthus* inducible by isopropyl-β-D-thiogalactopyranoside or vanillate. *Journal of Bacteriology* **194**:5875–5885. **PubMed:** [22923595](http://www.ncbi.nlm.nih.gov/pubmed/22923595) **DOI:** [10.1128/JB.01110-12](http://dx.doi.org/10.1128/JB.01110-12)

Julien B, Kaiser AD, Garza A. 2000. Spatial control of cell differentiation in *Myxococcus xanthus*. *PNAS***97**:9098–9103. **PubMed:** [10922065](http://www.ncbi.nlm.nih.gov/pubmed/10922065) **DOI:** [10.1073/pnas.97.16.9098](http://dx.doi.org/10.1073/pnas.97.16.9098)

Martin S, Sodergren E, Masuda T, Kaiser D. 1978. Systematic isolation of transducing phages for *Myxococcus xanthus*. *Virology* **88**:44–53. **PubMed:** [97862](http://www.ncbi.nlm.nih.gov/pubmed/97862) **DOI:** [10.1016/0042-6822(78)90108-3](http://dx.doi.org/10.1016/0042-6822(78)90108-3)

Nikolakakis K, Amber S, Wilbur JS, Diner EJ, Aoki SK, Poole SJ, Tuanyok A, Keim PS, Peacock S, Hayes CS, Low DA. 2012. The toxin/immunity network of *Burkholderia pseudomallei* contact-dependent growth inhibition (CDI) systems. *Molecular Microbiology* **84**:516–529. **PubMed:** [22435733](http://www.ncbi.nlm.nih.gov/pubmed/22435733) **DOI:** [10.1111/j.1365-2958.2012.08039.x](http://dx.doi.org/10.1111/j.1365-2958.2012.08039.x)

Pathak DT, Wei X, Bucuvalas A, Haft DH, Gerloff DL, Wall D. 2012. Cell contact-dependent outer membrane exchange in myxobacteria: genetic determinants and mechanism. *PLoS Genetics* **8**:e1002626.**PubMed:** [22511878](http://www.ncbi.nlm.nih.gov/pubmed/22511878)**DOI:** [10.1371/journal.pgen.1002626](http://dx.doi.org/10.1371/journal.pgen.1002626)

Wall D, Kaiser D. 1998. Alignment enhances the cell-to-cell transfer of pilus phenotype. *PNAS* **95**:3054–3058. **PubMed:** [9501214](http://www.ncbi.nlm.nih.gov/pubmed/9501214)**DOI:** [10.1073/pnas.95.6.3054](http://dx.doi.org/10.1073/pnas.95.6.3054)

Wall D, Kolenbrander PE, Kaiser D. 1999. The Myxococcus xanthus pilQ (sglA) gene encodes a secretin homolog required for type IV pilus biogenesis, social motility, and development. Journal of Bacteriology**181**:24–33. **PubMed:** [9864308](http://www.ncbi.nlm.nih.gov/pubmed/9864308)

Wei X, Pathak DT, Wall D. 2011. Heterologous protein transfer within structured myxobacteria biofilms. *Molecular Microbiology* **81**:315–326. **PubMed:** [21635581](http://www.ncbi.nlm.nih.gov/pubmed/21635581) **DOI:** [10.1111/j.1365-2958.2011.07710.x](http://dx.doi.org/10.1111/j.1365-2958.2011.07710.x)

Wei X, Vassallo CN, Pathak DT, Wall D. 2014. Myxobacteria produce outer membrane-enclosed tubes in unstructured environments. Journal of Bacteriology **196**:1807–1814. **PubMed:** [24391054](http://www.ncbi.nlm.nih.gov/pubmed/24391054) **DOI:** [10.1128/JB.00850-13](http://dx.doi.org/10.1128/JB.00850-13)

Wu SS, Kaiser D. 1995. Genetic and functional evidence that type IV pili are required for social gliding motility in *Myxococcus xanthus*. *Molecular Microbiology* **18**:547–558. **PubMed:** [8748037](http://www.ncbi.nlm.nih.gov/pubmed/8748037) **DOI:** [10.1111/j.1365-2958.1995.mmi_18030547.x](http://dx.doi.org/10.1111/j.1365-2958.1995.mmi_18030547.x)

Vassallo C, Pathak DT, Cao P, Zuckerman DM, Hoiczyk E, Wall D. 2015. Cell rejuvenation and social behaviors promoted by LPS exchange in myxobacteria. *PNAS* **112**:E2939–E2946. **PubMed:** [26038568](http://www.ncbi.nlm.nih.gov/pubmed/26038568) **DOI:** [10.1073/pnas.1503553112](http://dx.doi.org/10.1073/pnas.1503553112)

Vassallo CN, Wall D. 2016. Tissue repair in Myxobacteria: a cooperative strategy to heal cellular damage. *BioEssays* **38**:306–315. **PubMed:** [26898360](http://www.ncbi.nlm.nih.gov/pubmed/26898360) **DOI:** [10.1002/bies.201500132](http://dx.doi.org/10.1002/bies.201500132)

Vos M, Velicer GJ. 2006. Genetic population structure of the soil bacterium *Myxococcus xanthus* at the centimeter scale. *Applied and Environmental Microbiology* **72**:3615–3625. **PubMed:** [16672510](http://www.ncbi.nlm.nih.gov/pubmed/16672510) **DOI:** [10.1128/AEM.72.5.3615-3625.2006](http://dx.doi.org/10.1128/AEM.72.5.3615-3625.2006)
