# Supplementary material for: Infectious polymorphic toxins delivered by outer membrane exchange discriminate kin in myxobacteria
Source: eLife. 2017 Aug 18;6:e29397. doi: 10.7554/eLife.29397 (PMC5562445; doi:10.7554/eLife.29397)
Supplement: Supplementary file 3. — DOI: http://dx.doi.org/10.7554/eLife.29397.018 [file elife-29397-supp3.docx]

A

DK1622 SitA3 --MAAESLGKWVSL-WGTLLLASGCATLTPLSGHGVALGQGARVSHAGTLSS--SAGNTY

LILAB_13250 --MAAEPLGRWVAL-LGTMLLASGCATLTPLSGHGATLGQGARVSRAGTLSN--RAGNTY

A176_07430 -----MRAGVLV---VTLMLMATGCASTSPASRQQRGQGQGLPVTSTRPWNEAPSGGSSR

MYSTI_03282 -----MRAGLWM---LCAALFATGCAGVDASTRRTTFQQRGA--VPGEDGAD--ARADDE

MYSTI_04959 -----MRAGLWM---LCVALFATGCAGVDASARRTTFQQRGA--LPGEDGAD--ARADVE

LILAB_14775 -----MRAGLWV---VGVALLFMGCSGVESSTRRDTFHQRAG--LPGERWSV--SGEDPE

LILAB_02580 -----MRAGHWV---VGVALLLTGCAGVESSTRRETFHQRAA--LPGERWTA--SGEDAE

D187_007265 MPKSAERPWKWVALFLGSIVLATGCATVTTASGDGVLLDHGP--LVALSGSE--------

Q664_23910 --MFAEWARKWMPLVFGALVLSTGCITVMPPSGQGAF-----------------------

D187_005663 --MPAELARKWVALVLGAVVLSTGCITVTPSSGRGALLDRNPRAAPGLVVEI--RGDAQH

D187_005679 --MAAEMARKWVALVLGAVVLSTGCITVTPSSERGALLDLNPRAAPGLVGEG--HGDAQH

D187_002886 --MPAEMTRKWVALVLGAVVLSTGCITVTPPVGRGVLLDSNPRAASGLVGEV--RGDAQH

D187_002899 --MPAELKRKWVALVLGAVVLFTGCVTVTPPAGRGVLLDSNPRAASGLAGEI--RGDAQH

: :: ** .

DK1622 SitA3 LVASSAAASTDEDEEARLRRRRPGRASMGTAVIAASPADMAVLAVETGMLEVDAFEKLLV

LILAB_13250 LVASSAAASTDEDEEVRLRRRRSGRASMGTAVIAASPSDMAVLAVETGMLEVDAFEKLLV

A176_07430 VVAAEPLALSDSDDGARRSRRRAPRGD-GTDAGASSVGGIMQR----GSAHVDAWDALLS

MYSTI_03282 SDSQG-------EAEEPSSRPLRYRHPELREATRVGPAR---------RPRSDAFDALLR

MYSTI_04959 SDSQG--------EDGPSARPLRYRHPELRESTRVRPAR---------RPRSDAFDALLR

LILAB_14775 SEQEA-------EETDDSSRPPLHRRHMFRESVSLGVG---------GRPQ-DAFGVLLQ

LILAB_02580 YEPEA-------EAAEGASRQPLHRRHLFRDGMLLGGA----------RGQEDEFDVLLR

D187_007265 -----------------------------LKVALASPTEIVMRAVPGGAVRADAFDYLLL

Q664_23910 -------------------RHQGERVR-GTRMAMVSPAGMAVRAVAGGARQVDAFEHLLL

D187_005663 MATGS-------NAPARLLRRRGERVQ-GTEVAMVSPAEMAVHAVASGAVQVDAFEYLLA

D187_005679 AATGA-------NAPTRLLSRRGERVQ-GTKVAMASPAEMAVRAVASGARQADAFESLLL

D187_002886 VDTGA-------NAPARLLRRRGARVQ-STQVAMVSPAEMAVRAVASGARQADAFEYLLL

D187_002899 AATEA-------NAPARLLRRRGERVQ-STQVAMVSPAEMAVRAVASGARQADAFEYLLL

* : **

DK1622 SitA3 LAGLGDTDVLPPRVNPFTPEEADRLLALLVETPVTLGNFPPRMAVGHLLREVLEGGEVSR

LILAB_13250 LAGLGDTDVLPPRVNPFTPEEAARLLVLLVETPVTLGSFPPRMAVGHLLREVLEGGEVSR

A176_07430 RAGIDAQDARPIAGGSLPPIQAARLLKVLLSKPVTLAQFPARLAVGHLLRRALEEGELSR

MYSTI_03282 AAGLEEQDSRPVAGSSLTPTHAARLLNVLLRRDVTLGQFPARVAVGFMLREVLATGEVSR

MYSTI_04959 DAGLEEQDSRPVVGSSISPSHAARLLNVLLGKDVTLGQFPARVAVGFMLREVLVTGEVSR

LILAB_14775 NAGLEERDTRPVAGITLSPTHAARLLQTLLEKDVTLGQFPSRLAVGFMLREVLATGEVSR

LILAB_02580 NAGLEGRDARPVKGSALTPKHAARLLQALMGRDVTLGQFPARLAVGFMLREVLATGEVSR

D187_007265 LAGLDNVSEELPRDAILTPQKAALVLAGLLRKPVTPGSFPPRMAVCHLLREVVAGGSVSR

Q664_23910 LAGLDNVNDEPPRGAPLTPQEAARLLAVLLTKPVTLGSFPPRMAVCHLLREVLERGEVSR

D187_005663 LAGLDNVNDEPPRGAPLTPQEAARVLTVLMNKPVTLGSFPPRMAVCHLLREVLEGGDVSR

D187_005679 LAGLDNVNDEPPRGAPLTTEEAARVLAVLLNKPVTLGSFPPRMAVCHLLREVLDGGAVSR

D187_002886 LAGLDNVNDEPPRGAPLTPEEAARVLAVLLNKPVTLGSFPPRMAACHLLREVLAGGEVSR

D187_002899 LAGLDNVNDEPPRGAPLTPEEAARVLAVLLNKPVTLGSFPPRMAACHLLREVLAGGEVSR

**: . :.. * :* *: ** ..**.*:*. .:** .: * :**

DK1622 SitA3 AELLRRVARFQGVAVLRPDGYLAWVRNGRTQQRVAEVAWKDGAFRAHGFELGRFYGSQGG

LILAB_13250 AELLRRVDRFQGVAVLRPDGYLAWVRNGRTQQRVSEVEWREGAFRAHGFELGRFYGSRGG

A176_07430 AELLRQVERFSGVAVLRPDGCLAWVRSGKTQQRVAPVEWRDGGFRAHGFELGRFYDGRTG

MYSTI_03282 AELVRRVERFSHVAVLRPDGCLAWVLSGRTQQRVGEVEWEDGAFRAGLFELGVFYSGKGG

MYSTI_04959 AELARRVERFSGVAVLRPDGCLAWVLSGRTQQRVGEVEWKDGAFRAGLFQLGAFYSGKSG

LILAB_14775 VELDRRVERFRHLAVLRPDGCLAWVRSGRTQQRVAPVEWKDGAFRAHGFELGRFYDGRTG

LILAB_02580 AELVRRVERFRHLAVLRPDGRLAWVRSGRTQQRVAPVEWREGAFRAHGFELGRFYDGRTG

D187_007265 EELSRRVERFKRVAVLRPDGYLAWALNGRTQQKVGPVAWKDEAFRAGPFELGRFYSSHGG

Q664_23910 EALLHRVERFRRVAVLRPDGYLAWTLDGHTQQKVGPVEWKEEAFRAGPFELGRFYTVSGW

D187_005663 EELLRRVERFKSVAVLRPDGYLAWTLNGRTQQKVGPVEWKEEAFRAGSFELGRFYTVSGW

D187_005679 EELLRRVERFKTVAVLRPDGYLAWTLNGRTQQKVGPVEWKEEAFRAGSFELGRFYTVSGW

D187_002886 EELLRRVERFKGVAVLRPDGYLAWTLNGRTQQKVGPVEWKAEAFRAGSFELGRFYIVQGW

D187_002899 EELLRRVERFKGVAVLRPDGYLAWTLNGRTQQKVGPVEWKEDAFRAGSFELGRFYIVKGW

* ..* ** :******* ***. .*.***.*. * * .*** *:** **

DK1622 SitA3 IFRLLDAQFRELNEGA-YAEVYDDADVVSRTLDGAEDAFVELYHALGHLF-TYPTDSILA

LILAB_13250 VFRLLDAQFRELNEGA-YAEVYDDADVVSRTLDGAEEAFVELYHALGRFF-TYPTDSILA

A176_07430 VYRLLDDALREVDGRP-LADVHDDADYVGRTLDGAEAAFVKLALSVGHFL-TYPLDSLAA

MYSTI_03282 VFRQLNSRLEEVRGGA-YADVHDDADYLSRSLDGAEEAFVGLALAVGKFFSSTHEENLEA

MYSTI_04959 VFREMGSSLEELRGGA-YADVHDDADYLSRSLDGAEEAFVGLALAVGKFFSSSPEENLEA

LILAB_14775 VFRLLNEELREENGFP-IADVHDDADVISRTLDGAEEAFVGLALAVGKFFSTSPADNLAA

LILAB_02580 VFRLLNDELREESGFP-LADVHDDADVVSRTLDGAEEAFVGLALAVGKFFSTSPAESLAA

D187_007265 VFRSADAQLRPIMKEPPLAEEYDDADYISRTLDGAEESFVELYHAMGQLL-THPLDSLAA

Q664_23910 VFRQADAQLRPVPHGPGLAEVYDDADAINRSLEGAQESFVELYHSMGQLL-SHPSDSIAA

D187_005663 VFRQADAQLRPLMEGPPLAEVYDDADYIGRSLDGAEEAFVEMYHAMGQLL-TRPLDSIAA

D187_005679 VFRQADAQLRPLMQGPGLAEVYDDADYIGRSLDGAEDAFVGLYHAMGQLL-TRPLDSLAA

D187_002886 VFRQADAQLRPVMQGPGLAEVYDDADYLGRSLDGAEEAFVELYHVMGQLL-TRPLDSLAA

D187_002899 VFRQADAQLRPVMQGPGLAEVYDDADYLGRSLDGAEEAFVGLYHAMGQLL-TRPLDSLAA

::* . : . *: :**** :.*:*:**: :** : :*.:: : :.: *

DK1622 SitA3 LRHLPAGVAALIASSPEYWERFRYMTAGEQMKAVAKLSTTLLIAGGSANGVTRTLTSAMG

LILAB_13250 LRHLPAGVAALIASSPKYWERFRYMTAGEQMKAVARLSTSLLIAGGSANGVTRTLTSAIG

A176_07430 LKNLPAGVAALIASSPEYFERFRYMTRGEQIQVVSELATNLVLTTGTATATTRTVTGALA

MYSTI_03282 FRQMPAAVVALIKSSPEYLERFRYMTRGEQIQAVSRMVTNVIATWGTASTTTRTLQGA-A

MYSTI_04959 FRQMPAAVVALIKSSPEYLERFKYMTRGEQIQAVSKMVTNLIATWGAASSTTRTLQGA-T

LILAB_14775 LREMPAAVVALLESSPEYLERFRYMTRGEQVQAVSKLVTNLIATWGTVSAAARTLGGT-G

LILAB_02580 LRELPAAVVALLKSSPEYLERFRYMTRGEQVQAVSKLVTNLIATWGTASAATRTLEGT-A

D187_007265 LQRLPAGVAALIASSPDYFERFRYMTRGEQVKALSKLMTQLFVTFGAVKGTTSTLTRTFS

Q664_23910 LKHLPAGVAALIASSPQYLERFRYMTHGEQVKAVSKLLTHLLVTFGTAGGTTSTLTRAMG

D187_005663 LRHLPAGVAALIASSPEYLERFRYMTRGEQVKATSKLLTYLLVTFGTAGGTTSTLTRAVG

D187_005679 LRHLPAGVAALIASSPEYLERFRYMTRGEQVKATSTLLTHLIVTFGTAGGTTSTLTRAVG

D187_002886 LQHLPVGVAALIASSPEYLERFRYMTRGEQVKATSKLTTHLLLAFGTAGGTTSTLTRAVG

D187_002899 LQHLPAGVAALIASSPEYLERFRYMTRGEQVKATSKLLTHLLVTFGTAGGTTSTLTRAVG

:. :*..*.**: ***.* ***.*** ***::. : : * :. : *:. .: *: :

DK1622 SitA3 GAEAMVPILSLSAQGALAIQRVAVPVGKMATVVGAGVGTIVVLSQAAGSGGG----PKLK

LILAB_13250 GAEAMVPVFSLSAQGALAIQRVAVPVGKVATAVGDGVGGVYVLSTAGDTPGG--------

A176_07430 GADATVPVLALSAQGALTFERVAAPVGRAAAVLGGGPGAVIILQRASTTAQG----AG-P

MYSTI_03282 LATAEVPVLSVSAQGALALERVAVPVGRTAAVLSGGPGAAIILQRAGTEAKQ----GG-P

MYSTI_04959 LATAEVPVLSVSAQGALALERVSVPVGRAAAVLNGGPGAAIVLHRAGTAAQQ----GG-P

LILAB_14775 FATAEVPALVLAADGTVAMRLVSASAGRAAGVLSGGPGAAIILQRAGEGGGS----SATP

LILAB_02580 LATAEVPALVLSAQGTVAMRLVAAPVGRAAAVLSGGPGAAIILQRAGTAAKD----GA-P

D187_007265 GTEATVPVLSLSAEGALMVERVAVPVGKVATVLSGGPWAAIIVQQAHGAATG-------A

Q664_23910 GLEATVPVLSLSAEGLLVVERVVVPVGKVATVLSGGPGAAIILQRANASGES----PS-P

D187_005663 GLEATVPVLSLSAEGLLVVERVVVPVGRAATVLSGGPGSAIILYQVGSGGGG---EGGGE

D187_005679 GLEASVPVLSLSAEGLLMVERVVVPVGKTATVLSGGPGATIILQRASTGTNE----ARPS

D187_002886 ELEVTLPVLSLSAEGLLVMRMVAVAAGTVTTTLEVGVGSVSILHMASRGQRANGVDPPRP

D187_002899 GLEVTLPVLSLSTEGLLAVRTVTVAAGTVTTTLEVGVGTVSILHMASRDDQP----SGSK

. :* : ::::* : . * ...* : .: * :: .

DK1622 SitA3 GSLTGRKTTPGPHDKDPA-NIKSIQRENESAEILAENGYHVEQNPAPKP-NGKEPDYRIN

LILAB_13250 ----NGTAAPSSSREGSV-RDYVRMGDNGNGIVATMDGEGVVEMAIEAPKGSAIRGSELF

A176_07430 SR-GPGRWGPAKESMKPP-ARRYQEQISGHSADDAYWVGGMSSQAGGVKFDGFKDGVLLE

MYSTI_03282 AK-EPGEWGPSEEKGASARARAYQEQISGRSYDDAYWVGGVGRKSGGTKFDGFEDGVLLE

MYSTI_04959 SEGGPGRWESASESMSDA-SREYQAQVTGAPIGKVYNVEGV-------RFDGFNLGVLLE

LILAB_14775 PA-GPG--GMKSYSSFKS-FKR----AMGPAGPGKEWHHIVEQTDGNV--GRFGPKAIHN

LILAB_02580 SK-GPGQWGPAKESMSPR-ARRYQEQIAGHSADEAYWVGGVGRDRGGVKFDGFDAGVLLE

D187_007265 SEASRGQMRPAAEELLQQATRLPPERLGGYRIFGNKGLVGQTFQ---------RNILLIE

Q664_23910 AA-GPGRWGPARESMSSR-ARAYQEQISGHSADEAYWVGGTSAKDGGVRFDGFKDGVLLE

D187_005663 ERTGGTNWKPGPNDLD---WRGVGKGVPE-ALDEAFKRTGVSRE---------------E

D187_005679 ARDYPQDYASARTQNHS--ARFSSEREARSLARQKIGKDPVEFE----------SGKLRS

D187_002886 VR-SPNRAEPRIEDGNP--QEGWQHIEARHITGTHPNGQGDLFAPGTSRAQLLEAAKDIV

D187_002899 TK-NP--YSSLTRTQLEKSRRSFIKLIDEH------------------------EKKLLE

DK1622 SitA3 GE---YADCYAPNGRRVRNMWDYVR--E-SKVESGQ--ADRIVMNLEDT-----PVPVAE

LILAB_13250 RE---MMEHFGGNVRAIRGNWRYGDNLGEVNTLVSQ--GVRLEDAVKRT-----WTARRA

A176_07430 AKGPGYA-KFFDELEPK--EWFLHS--G-AKDLVKQ--ADRQIRAVRGQGLRIEWHVAEA

MYSTI_03282 AKGPGYAEFFETNLTPK--GWFEAS--GKARDLVDQ--ALRQIGKVKGKGIPIEWHIAEK

MYSTI_04959 TKGPGYA-KFLKDGSFR--AWFRG-----ADGMLDQ--ARRQFEVARGT--PIHWHFAER

LILAB_14775 TE--NIV-ALDKELHTRVSAFYSSK--N-VSATGSD--AF-TVR---------QWLRAQS

LILAB_02580 AKGPGYA-RFFEELDPK--RWFKNS--G-AKALGEQ--ALRQVKAAKGM--PIRWHVAEE

D187_007265 ADKGAALSRLIAALE--------------AEARAAG--ASRLSITGHAVINKGFLKPEIA

Q664_23910 AKGPGYANKFFDTLEPR--PWFKHS--G-AKALVDQ--AERQLRAARTTGTPIRWHVSEE

D187_005663 FAVTKWGRDKLGKSAPV--EWRAPN--G-AEVNIDM----GHIKNGPSVPHVGFQTPGKR

D187_005679 RDG----------------KWQYRA--R-PEDLKGHRPGDTP-------------HVHLE

D187_002886 KNGNRIS-------EPD------------NGNRISE--PDRRIQVFQMK-------VKLN

D187_002899 YRTDPFANDNLGKL----------------ENQPPE--NQRKIISGRIE----EVEAQLT

DK1622 SitA3 VREQFLKYPIQGLKEVLGIKDGKVSLIYP

LILAB_13250 AEYGFSRAVVTEVQGAPGAYKFIQVLFER

A176_07430 KAAAAIRALLHE-AGVEGVKVLHTPAL--

MYSTI_03282 HAADAIRKLLRS-NNAREISIIHTPAL--

MYSTI_04959 EVASAVRDLFRT-EGLGAIKVVHTPIAP-

LILAB_14775 YEAQ------RE-FGLRALENVRKGLWR-

LILAB_02580 TAVPALRKLLQD-RGIKGIEVVFTPPL--

D187_007265 QRFGFELRLINK-DTIELVKELRP-----

Q664_23910 KVANAIRRLFKL-ENVAGIEVVYTPAL--

D187_005663 GSGGAIRGHILL-DDVPYNR---------

D187_005679 RLDPKTGEVLEN-WHLRW-----------

D187_002886 GVRDLVRVIIDS-DDANRVITMFPVRGG-

D187_002899 KQRGELRKVEEALRALNGGG---------

B

| Organism | Locus tag | Pfam domain | Probability | E-value |
| --- | --- | --- | --- | --- |
| *Myxococcus fulvus* | LILAB_13250 | PF15540, Ntox47 | 98.0 | 5.4E-06 |
| *Myxococcus sp.* | A176_07430 | PF15648, Tox-REase-5 | 100.0 | 1.4E-33 |
| *Myxococcus stipitatus* | MYSTI_03282 | PF15648, Tox-REase-5 | 100.0 | 4.5E-33 |
| *Myxococcus stipitatus* | MYSTI_04959 | PF15648, Tox-REase-5 | 100.0 | 2.6E-30 |
| *Myxococcus fulvus* HW1 | LILAB_14775 | PF12639, Colicin-DNase | 94.5 | 0.088 |
| *Myxococcus fulvus* HW1 | LILAB_02580 | PF15648, Tox-REase-5 | 100.0 | 2.2E-31 |
| *Cystobacter fuscus* | D187_007265 | PF00583, acetyltransferase | 85.0 | 2.5 |
| *Cystobacter violaceus* | Q664_23910 | PF15648, Tox-REase-5 | 100.0 | 1.7E-31 |
| *Cystobacter fuscus* | D187_005663 | PF15540, Ntox47 | 100.0 | 7.9E-39 |
| *Cystobacter fuscus* | D187_005697 | PF03413, PepSY | 88.4 | 0.9 |
| *Cystobacter fuscus* | D187_002886 | PF15648, Tox-REase-5 | 99.2 | 1.3E-12 |
| *Cystobacter fuscus* | D187_002899 | PF10458, Valyl tRNA synthetase | 95.2 | 0.92 |

**Supplementary file 3**. Alignment of representative SitA toxins and predicted functions of their CTD. (A) The N-terminal ~100 residues of SitA homologs are variable and tend to be species-specific (colored shading indicates similar N-terminal region), followed by a relatively conserved central domain and then contain polymorphic C-terminal toxin domains. This alignment shows representative SitA3 homologs generated with MUSCLE (Edgar, 2004). Sequences following the last conserved central domain residue (gray highlight) in each sequence was subject to functional domain analysis by HHpred (Soding et al., 2005). (B) Tabulation of the Pfam (Finn et al., 2016) domains with the highest returned scores from HHpred.

References

Edgar RC. 2004. MUSCLE: multiple sequence alignment with high accuracy and high throughput. Nucleic Acids Research **32**:1792–1797. **PubMed:** [15034147](http://www.ncbi.nlm.nih.gov/pubmed/15034147) **DOI:** [10.1093/nar/gkh340](http://dx.doi.org/10.1093/nar/gkh340)

Finn RD, Coggill P, Eberhardt RY, Eddy SR, Mistry J, Mitchell AL, Potter SC, Punta M, Qureshi M, Sangrador-Vegas A, Salazar GA, Tate J, Bateman A. 2016. The pfam protein families database: towards a more sustainable future. Nucleic Acids Research **44**:D279–D285. **PubMed:** [26673716](http://www.ncbi.nlm.nih.gov/pubmed/26673716) **DOI:** [10.1093/nar/gkv1344](http://dx.doi.org/10.1093/nar/gkv1344)

Söding J, Biegert A, Lupas AN. 2005. The HHpred interactive server for protein homology detection and structure prediction. Nucleic Acids Research **33**:W244–W248. **PubMed:** [15980461](http://www.ncbi.nlm.nih.gov/pubmed/15980461) **DOI:** [10.1093/nar/gki408](http://dx.doi.org/10.1093/nar/gki408)
